# Production of Lipopeptide Biosurfactant by a Hydrocarbon-Degrading Antarctic *Rhodococcus*

**DOI:** 10.3390/ijms21176138

**Published:** 2020-08-26

**Authors:** Syahir Habib, Siti Aqlima Ahmad, Wan Lutfi Wan Johari, Mohd Yunus Abd Shukor, Siti Aisyah Alias, Jerzy Smykla, Nurul Hani Saruni, Nur Syafiqah Abdul Razak, Nur Adeela Yasid

**Affiliations:** 1 Department of Biochemistry, Faculty of Biotechnology and Biomolecular Sciences, Universiti Putra Malaysia, Serdang, Selangor 43400, Malaysia; syahirhabib@gmail.com (S.H.); aqlima@upm.edu.my (S.A.A.); mohdyunus@upm.edu.my (M.Y.A.S.); hanisaa17@gmail.com (N.H.S.); syaffy27@gmail.com (N.S.A.R.); 2 Department of Environment, Faculty of Forestry and Environment, Universiti Putra Malaysia, Serdang, Selangor 43400, Malaysia; wanlutfi@upm.edu.my; 3 Institute of Ocean and Earth Sciences, C308 Institute of Postgraduate Studies, University of Malaya, Kuala Lumpur 50603, Malaysia; saa@um.edu.my; 4 Institute of Nature Conservation, Polish Academy of Sciences, Mickiewicza 33, 31-120 Kraków, Poland; jerzysmykla@yahoo.com

**Keywords:** surface-active lipopeptide, biosynthetic gene clusters, fellfield soil, Antarctica

## Abstract

Rhodococci are renowned for their great metabolic repertoire partly because of their numerous putative pathways for large number of specialized metabolites such as biosurfactant. Screening and genome-based assessment for the capacity to produce surface-active molecules was conducted on *Rhodococcus* sp. ADL36, a diesel-degrading Antarctic bacterium. The strain showed a positive bacterial adhesion to hydrocarbon (BATH) assay, drop collapse test, oil displacement activity, microplate assay, maximal emulsification index at 45% and ability to reduce water surface tension to < 30 mN/m. The evaluation of the cell-free supernatant demonstrated its high stability across the temperature, pH and salinity gradient although no correlation was found between the surface and emulsification activity. Based on the positive relationship between the assessment of macromolecules content and infrared analysis, the extracted biosurfactant synthesized was classified as a lipopeptide. Prediction of the secondary metabolites in the non-ribosomal peptide synthetase (NRPS) clusters suggested the likelihood of the surface-active lipopeptide production in the strain’s genomic data. This is the third report of surface-active lipopeptide producers from this phylotype and the first from the polar region. The lipopeptide synthesized by ADL36 has the prospect to be an Antarctic remediation tool while furnishing a distinctive natural product for biotechnological application and research.

## 1. Introduction

Antarctica is generally perceived as one of the most pristine environments on Earth. Nevertheless, Antarctica’s environments are not immune to impacts from human activities occurring both globally and locally within the region. In fact, growing research interest and exploration have already substantially altered some areas, particularly in regions with a high concentration of multinational activities and presence of research stations. Fuel spills and hydrocarbon pollution are one of the most frequent effects of human impacts. The contamination of hydrocarbon components in the Antarctic has caused great concern as their environmental recovery rate is expected to be very slow, therefore having the potential to cause pronounced and long lasting environmental impacts [1,2].

Bioremediation processes hold a great promise in exploiting the ability of polar microorganisms to metabolize hydrocarbon substrates. However, one of the major obstacles that affect biodegradation efficiency is the low availability of the hydrocarbon substrates due to their low water solubility and strong binding to soil particles. The extremely low temperatures in the Antarctic worsen the situation as it increases the viscosity of the hydrocarbons [3]. Production of biosurfactants is one of the strategies employed by cold-adaptive Antarctic strains to increase the bioavailability of hydrocarbon substrates and facilitates their uptake into the cells [4,5]. These biosurfactants can be classified into various categories such as glycolipids, phospholipids, neutral lipids, polymeric surfactants and lipopeptides [6]. Acknowledged for their excellent surface activity, biosurfactants reduce the surface and interfacial tension to promote more interactions and mixing between immiscible liquid phases such as oil and water interfaces [7].

The awareness towards environmental protection, rigorous ecological policies and simultaneous growth in consumer’s demand for bio-based products has greatly increased. Thus, the “green” synthesis of surface-active agents from cold-adapted microorganisms can be considered as safe alternatives to their synthetic counterparts. The microbial surfactants have many advantages compared to their chemically-synthesized equivalents as they are environmentally-friendly, highly biodegradable, highly selective and specific at extreme temperatures, pH and salinity, less toxic and non-hazardous [8,9]. Owing to these features, biosurfactants are extensively used in many commercial applications such as cosmetics, personal care, textile manufacturing, food processing, agricultural formulations, medicinal and biomedical industries, soil remediation, hydrocarbon decontamination and oil recovery [6].

Bacteria from the genus *Rhodococcus* are considered as naturally ubiquitous in the Antarctic environment due to their frequent occurrence and dominance in both pristine [10] and anthropogenic-polluted ecosystems [4]. The excellent ecological properties of *Rhodococcus* spp. such as wide catabolic diversity, high persistence in low oxygen and nutrient conditions, great adaptation to hydrophobic substrates and high resistance to environmental stresses earns them a reputation as an outstanding microbial vehicle to remediate petroleum hydrocarbons and heterocyclic compounds [11]. Several prevalent members of this genus such as *Rhodococcus erythropolis*, *Rhodococcus opacus*, *Rhodococcus equi*, *Rhodococcus rhodochrous*, *Rhodococcus ruber*, and *Rhodococcus fascians* also produce biosurfactants in response to the presence of liquid hydrocarbons in the growth medium [4,5,12].

*Rhodococcus* biosurfactants are largely cell-bound glycolipids with the generic link of trehalose sugar, commonly known as trehalolipid [13,14]. Hence, the discovery of an unusual production of a lipopeptide in the course of this study was intriguing although novel lipopeptide from this genus has been reported twice [15,16]. Studies have reported that the polar microbial diversity is much richer than previously thought, comprising a number of prominent producers of bioactive natural products such as *Actinobacteria* and *Proteobacteria* with advanced and novel survival machinery [17,18]. Moreover, these psychrophiles and psychrotrophs may also possess peculiar and evolved survival mechanism that could be explored through bioprospecting, such as the maintenance of membrane fluidity through the synthesis of unique lipids [19]. In most occasions, such a mechanism is typically regulated via the polyketide synthases (PKS) and non-ribosomal peptide synthetase (NRPS) gene clusters [20,21]. These clusters of biosynthetic genes are regarded as one of the most progressive microbial genes, due to their frequent occurrences in horizontal gene transfer [22]. For this reason, it is likely that cold-adapted bacterium such as *Rhodococcus* sp. ADL36 can also harbor distinctive biosynthetic gene clusters (BGCs).

In the present study, we highlighted for the first time the potential of a bacterial strain from *Rhodococcus* phylotype isolated from the Antarctic fellfield soil for synthesizing tensio-active lipopeptide. The surface-active molecules produced by the strain ADL36 were analyzed through chemical characterization while the BGCs related to lipopeptide synthesis were mined through genomic exploration. The employment of the integrated in vitro and in silico approaches allows the comprehension of the genetic basis of the mechanism involved in the production of the lipopeptide while understanding its biochemical properties.

## 2. Results and Discussion

### 2.1. Screening for the Synthesis of Biosurfactant

#### 2.1.1. Hydrophobicity Assay

The evaluation of cell adherence towards hydrophobic substrates such as hydrocarbons is widely considered as an indirect method for the screening of biosurfactant-synthesising bacteria. The method lies in the notion of the attachment of cells to the hydrophobic compounds by synthesising the surface-active compounds. BATH assay conducted in this study revealed an average cell adhesion value of 70.01 ± 0.29 for diesel oil and 68.13 ± 2.27 for used motor oil. Positive cell hydrophobicity acts as an indicator of a potential biosurfactant producer [23,24].

#### 2.1.2. Drop-Collapse Test

The drop-collapse test is a sensitive and rapid assay to screen biosurfactant-producing bacteria [25]. The principle behind this test is that cell-free supernatant droplets will collapse and expand on the oil surface if it contains biosurfactants whereas water droplets will remain beaded due to the polar water molecule that repels against the hydrophobic surface of the oil. The collapsing behaviour is due to the reduction of both surface and interfacial tension between the oil surface and the supernatant [26]. The advantage of this method is that only small amounts of sample are needed and it does not involve any specialised equipment. The bacterial supernatant of ADL36 showed positive collapse while the control (distilled water) remained stable, indicating the absence of biosurfactant (Figure S1 (Supplementary Materials)). Strain ADL36 showed a near-complete collapse with a diameter of 1.07 ± 0.1 cm.

#### 2.1.3. Oil Spread Test

The oil spreading test is an indirect measurement of the surface activity where a larger diameter indicates a higher surface activity of the testing solution. According to Youssef et al. [27], the area of oil displacement in this approach corresponds to the concentration of the biosurfactant solution, although no quantitative study has been conducted on biosurfactants concentration against the oil displacement activity. Akin to drop collapse method, oil displacement technique is rapid, easy to perform, requires only a small volume of samples and does not involve any specialised apparatus. A significant oil clearance zone indicates a positive oil displacement activity for strain ADL36. Contrary to the drop collapse method, oil spreading tests can be considered as more sensitive in detecting low concentrations of biosurfactants even though both methods are reliable to test biosurfactant synthesis as they eliminate false negatives (those with low concentrations of biosurfactants) [27]. The results show that supernatant broth from strain ADL36 was able to displace the same amount of used motor oil although it exhibited a lower displacement activity than the chemical supernatants sodium-dodecyl-sulphate (SDS) and Triton X-100 (Figure S2 (Supplementary Materials)). No displacement activity was observed using distilled water.

#### 2.1.4. Microplate Assay

This procedure is a simple qualitative test and has been developed and patented by Vox and Cuttingham [28]. This assay relies on the change in optical distortion that is caused by surfactants in an aqueous solution. The bacterial supernatant showed the presence of biosurfactant as the grid image was distorted due to the wetting of the wall edges (Figure S3 (Supplementary Materials)). This caused the surface of the biosurfactant solution in the well to appear concave and took the shape of a diverging lens.

#### 2.1.5. Emulsification Index (E_24_)

Emulsification capacity is one of the essential assessments to evaluate the production of biosurfactant by bacterial cells. The principle lies in the formation of the emulsion layer between the oil and supernatant. The greater the emulsion layer formed, the higher the production of biosurfactant. Strain ADL36 was found to produce biosurfactants by the capacity to emulsify the diesel oil with E_24_ of 45.33 ± 4.98% and used motor oil with E_24_ of 47.45 ± 2.35% (Figure S4 (Supplementary Materials)). While E_24_ is highly considered as the most extensively used method to measure the surface activity due to practical ease [5], a number of synthesised biosurfactants can either stabilise or destabilise the emulsion [29]. This can cause inefficiencies in the identification of surface-active compounds during the screening processes.

### 2.2. Biosurfactant Stability Test

The stability test was monitored using two different approaches which awere E_24_ and surface tension (ST). Apart from emulsification activity, the assessment of surface activity is considered as a good indicator for biosurfactant synthesis. The broad use of ST measurement using the du Noüy ring method [30] is becauseo if its accuracy, ease of use and fairly rapid measurement of surface and interfacial tension although specialised equipment is compulsory. Several drawbacks of this approach include the requirement of large sample volume and a limited range of concentrations that can be analysed without dilutions [26]. The stability of biosurfactants in different conditions directly affects the applicability of the surface-active agents in the environment.

**Figure 1:**
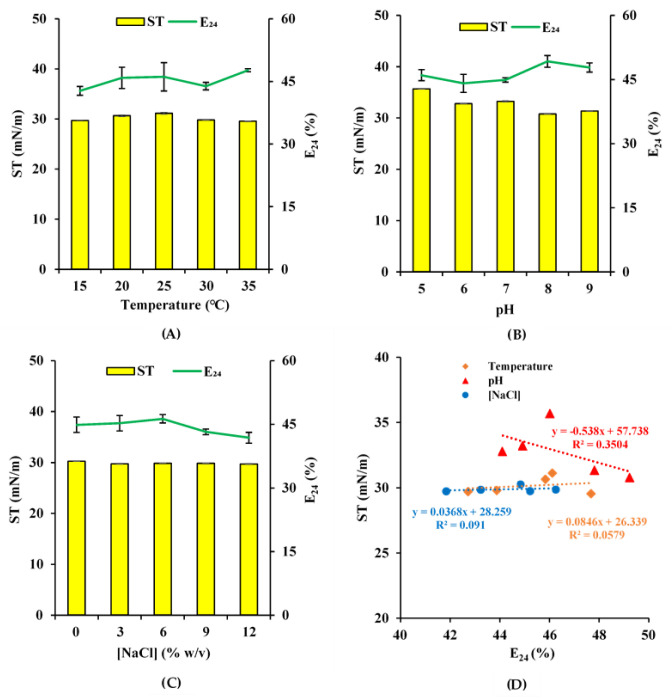
The stability of biosurfactants produced by *Rhodococcus* sp. ADL36 at different: (**A**) temperature; (**B**) pH and (**C**) salinity. (**D**) No significant correlation was found between emulsification index (E_24_) and surface tension (ST) for temperature, pH and NaCl concentration.

The highest E_24_ for biosurfactants produced by *Rhodococcus* sp. strain ADL36 was recorded at 35 °C (47.68 ± 0.34%) while the ST showed no significant differences among the examined temperatures (*p* < 0.05) (Figure 1A). As temperature is one of the most fundamental factors that significantly affects the growth of microorganisms and their biosurfactant synthesis, any rise or fall in the emulsification or surface activity under extreme temperatures could be caused by some structural alteration in the biosurfactant molecule. Strain ADL36 showed low and irregular emulsification capacity within the analysed temperature compared to previous reports where *Rhodococcus* spp. showed higher E_24_ from low to high temperatures (100 °C) [31,32]. However, ADL36 exhibited a stable ST value across the whole investigated temperature range.

Biosurfactants produced by strain ADL36 showed the highest stability at pH 8.0 (E_24_ = 49.24 ± 1.37%; ST = 30.79 ± 0.04 mN/m) (Figure 1B). The ADL36 cell-free supernatant showed lower emulsification capacity and high surface activity at the lower pH in contrast to the near-neutral pH. Kazemi et al. [32] observed that the biosurfactants produced by *Rhodococcus erythropolis* strain P6-4P was precipitated at low pH and exhibited high surface tension. Comparatively similar trends have been reported for the emulsification capacity of *Rhodococcus* sp. strain PML026 by White et al. [31] where low E_24_ has been observed from acidic to near-neutral conditions (pH 2, 4 and 6), highest at neutral pH and dropped at alkaline state (pH 10 and 12). Nonetheless, the same report also showed no correlation between E_24_ and ST as a low ST value was recorded at pH 2 while the highest surface activity occurred at pH 12. Tensio-active agents commonly produced by the *Rhodococcus* genus form different types of aggregates depending on the pH of the medium [33,34]. In basic conditions, the presence of electrostatic repulsions between the negatively charged carboxylate groups on the surfactant molecules complicates aggregation [35], leading to lower emulsification intensity.

The analyses of biosurfactant stability in the salinity gradient have demonstrated that the biosurfactant produced by the ADL36 maintains its ability to decrease the ST within the whole range of the investigated salinity up to 12% (*w*/*v*) of NaCl. On the other hand, gradual reduction of E_24_ values was observed in the higher salt concentrations (Figure 1C). Excellent stability of synthesised biosurfactant (cell-free supernatant) in a high concentration of salt was also reported previously [31,32]. The low ST measurement of ADL36 cell-free supernatant recorded within the whole range of temperature, pH and salinity tested indicates that the strain is a promising surfactant producer. No study on the thermal, pH and salinity stability for polar strains has been reported prior to this data. The pH stability for the supernatant broth of ADL36 shows a moderate negative correlation between E_24_ and ST (r_s_ = −0.592). Contrariwise, thermal and saline stability exhibited a weak positive correlation between the two parameters with the value of r_s_ = 0.241 and r_s_ = 0.302, respectively (Figure 1D). The correlation results between the two biosurfactant screening techniques in this work do not comply with previous reports [36,37], where a strong negative correlation was observed between emulsifying and surface activity (higher E_24_ resulted in lower ST). However, several studies have reported that strains with low ST value were not commonly adept for the formation of emulsion, suggesting the absence of correlation between the methods [38,39]. Cirigliano and Carman [40] interpreted the phenomenon as the reliance of microorganisms to certain conditions or media for the synthesis of either biosurfactant with emulsifier properties or ST reducing activity. This elucidation was supported by other studies [38,41] as they categorised biosurfactant into low molecular weight molecules, which lower ST at the air–water interface, and high molecular weight polymers, which have greater emulsifying activity (bioemulsifier). Accordingly, Uzoigwe et al. [7] strongly emphasised the need for a new screening method to distinguish between bioemulsifiers and biosurfactants. However, the high inconsistency observed in the STs and emulsion formation during the screening may be related to high variability in several other factors which includes carbon substrate (diesel) [5], concentrations of ions in culture medium [42], culture age, as well as environmental influences from the examined variables (pH, temperature and salinity).

### 2.3. Recovery of Crude Biosurfactant and Estimation of Carbohydrate, Protein and Lipid Content

The use of a different solvent system for extraction yields only a small difference in the amount of crude biosurfactant. The yield of the crude extract obtained using methyl-tert-butyl ether (MTBE) and chloroform-methanol was 0.34 and 0.31 g/L, respectively. The compositional analyses of the crude extract revealed that the biosurfactant synthesised by *Rhodococcus* sp. ADL36 might be a lipopeptide as the relative content of the protein and lipid was approximately 25% and approximately 64%, respectively. The extracted biosurfactant also contained a low concentration of carbohydrate (<3%).

### 2.4. Thin-Layer Chromatography

The partially purified biosurfactant recovered from the extraction (MTBE extract) process revealed a dark pink spot (R*_f_* = 0.372) when sprayed with ninhydrin indicating the presence of peptide moiety. The formation of brown/yellowish spots using iodine vapour suggests the presence of polar lipids with the R*_f_* value of 0.594 (Figure S5 (Supplementary Materials)). The absence of spots after the treatment with Molisch reagent implies the lack of carbohydrates in the sample. Previous reports demonstrated that the presence of red or pink spots on a thin-layer chromatography (TLC) silica plate using the combination of a solvent system containing *n*-butanol:acetic acid:water (4:1:1) and ninhydrin spray indicates the presence of lipopeptide molecules [15,43].

### 2.5. Fourier-Transform Infrared Spectroscopy Analysis

The FTIR spectroscopy was used to validate the functional groups and the chemical bond present in the unknown sample, hence elucidating the chemical nature of the biosurfactant. In this study, the presence of significant peaks in the FTIR spectrum for the biosurfactants produced by the Antarctic strain was assessed from 400–4000 cm^−1^ (Figure S6 (Supplementary Materials)).

Infrared spectroscopic analysis of the partially purified compound by using MTBE as the solvent of choice reveals several significant peaks across the spectrum. A strong and broad peak at 3290 cm^−1^ corresponds to the presence of -NH functional groups (amide A). Three sharp and slightly small peaks at 2925 cm^−1^, 2854 cm^−1^ and 1460 cm^−1^ indicated the presence of aliphatic bonds CH, CH_2_ and CH_3_, respectively. The sharp and intense peak at 1630 cm^−1^ most likely specifies the presence of amino acid zwitterion -C=O or amide carbonyl, a typical indication of a lipopeptide molecule. The absorption band at 1539 cm^−1^ infers the occurrence of an amide II band due to the stretching/banding of N-H bonds. A small peak at 1070 cm^−1^ designates the carbonyl stretching (C=O).

Similarly to MTBE, the employment of chloroform-methanol solvent system for the recovery of partially purified biosurfactant displays multiple significant peaks along the infrared spectrum. A relatively broad peak indicating the N-H stretching vibration was observed at 3282 cm^−1^. The occurrence of a sharp and narrow band at 2923 cm^−1^ demonstrates the presence of an alkyl chain (-CH_2_). An intense and strong band occurred at 1638 cm^−1^ which can be attributed to the CO-NH bend (governed by the stretching vibrations of both C=O and C-N groups), which verifies the presence of a peptide group in the biosurfactant molecule. An absorption band at 1535 cm^−1^ implies the presence of an amide II band due to C-N stretching vibrations with the combination of N-H bending. Other significant peaks observed at 1448 and 1374 cm^−1^ might correspond to C-H vibrations.

The occurrence of several N-H bonds stretching with the combination of several aliphatic groups across the FTIR spectra emphasise the nature of the lipopeptide surfactant. Similar FTIR absorption spectra for lipopeptide were previously reported in other studies [44,45]. Initially assumed as trehalolipid due to the general synthesis of this biosurfactant for this genus, the high number of amide functional groups in the IR analysis reveals the true nature of the studied biosurfactant as a lipopeptide. In addition, the lack of notable peaks at 1680–1750 cm^−1^ denotes the absence of a lactone ring, signifying that the lipopeptide produced by ADL36 is linear and not in a cyclic form. However, this conjecture may not be accurate as the extracted surfactants are not in their purified form, thus they may not reveal the accurate nature of the synthesised biosurfactants. To date, only two studies have recorded the synthesis of lipopeptides from the *Rhodococcus* sp. [15,16]. Interestingly, a minor yet critical resemblance was detected to the report of Peng et al. [15] where no precipitate was produced using the acid precipitation method towards the culture supernatant. This causes the authors to speculate that the extracted surface-active agent is likely distinct from other lipopeptides in previous studies.

### 2.6. Genomic Analyses and Anti-SMASH Prediction

The genomic data reveals a typical Rhodococci feature with an approximate size of 8,440,759 bp, a G + C content of 63.28%, 8462 protein-coding sequences (CDs), 6 rRNA and 82 tRNAs genes. The large size and high CDs of the draft genome are believed to be associated with the extensive actinobacterial chromosome linearisation and dependent to the host itself, which are isolated from an extreme niche (Antarctic soil) [46]. The annotated data also shows a high similarity with two other known genomes of *R. erythropolis* (PR4 and CCM2925) (Table 1). Among the Rhodococci species, *R. erythropolis* is widely known in xenobiotic mineralisation as numerous industrially and environmentally important enzymatic reactions have been described in various strains [47,48]. A 16S rRNA gene sequence of approximately 1500 bp (retrieved via RNAmmer 1.2) was found to be 100% identical with *R. erythropolis* PR4 (NR_074622) [49] and *Nocardia coeliaca* DSM 44,595 (NR_104776) [50].

Antibiotics and secondary metabolite analysis shell (anti-SMASH) predict clusters of genes which belong to known biosynthetic class, whereas the ClusterFinder algorithm (selected as an extra annotation feature) helps to seek out putative gene clusters of unknown function based on observed local protein domain frequencies [52]. Secondary metabolites prediction using both the anti-SMASH and the anti-SMASH-incorporated NaPDoS pipelines revealed the presence of several major BGCs such as PKS, terpene, saccharides, siderophores, ribosomally synthesised and post-translationally modified peptides (RiPPs) and a noticeably high number of NRPS (Table 2). The data obtained are in agreement with the observation of both Ceniceros et al. [53] and Doroghazi and Metcalf [54], where high numbers were predicted for NRPS clusters compared to the PKS clusters in Rhodococci genomic sequences. Nonetheless, because the analysis was performed on drafted genome sequences, the ratio of core BGCs like NRPS to PKS might be overestimated (contigs splitting).

NRPS are large multimodular enzymes that make up of repeated modules which incorporate in a specific manner (activation, modification and condensation) of a single amino acid into the peptide backbone [55]. An essential NRPS module is comprised of an adenylation domain (A) that identifies and activates a specific amino acid substrate into an aminoacyl adenylated intermediate, a peptidyl carrier protein (PCP) or thiolation (T) domain that binds covalently to the activated residue via thioester bond and a condensation domain (C) which is responsible for elongation of the peptidyl chain. Different types of C domains catalyse different types of condensation reactions. The assembled and final peptide chain is terminated and released from the modular complex via a thioesterase domain (TE) [56].

While most modules were set in motion via the A domain, a distinct C domain subtype called the *C*-starter domain is the first domain in several NRPS assembly lines. This starter domain drives the acylation of the first amino acid (with the presence of *β*-hydroxy or *β*-amino fatty acid) of the peptide moiety, thereby synthesising a lipopeptide [55,56]. Among the eight NRPS clusters found in ADL36, seven had a *C*-starter domain (NRPS-1, −3, −4.1, −4.2, −6, −8, −67), suggesting the synthesis of a lipopeptide. Three of the clusters that began with the unique *C*-starter domain (NRPS-1, NRPS-3 and NRPS-8) displayed similarities to the siderophores heterobation A/S2 (100%), erythrochelin (57%) and coelichelin (27%). Another two clusters (NRPS-4, −67) did not show any similarities to any known gene cluster in the database.

**Figure 2:**
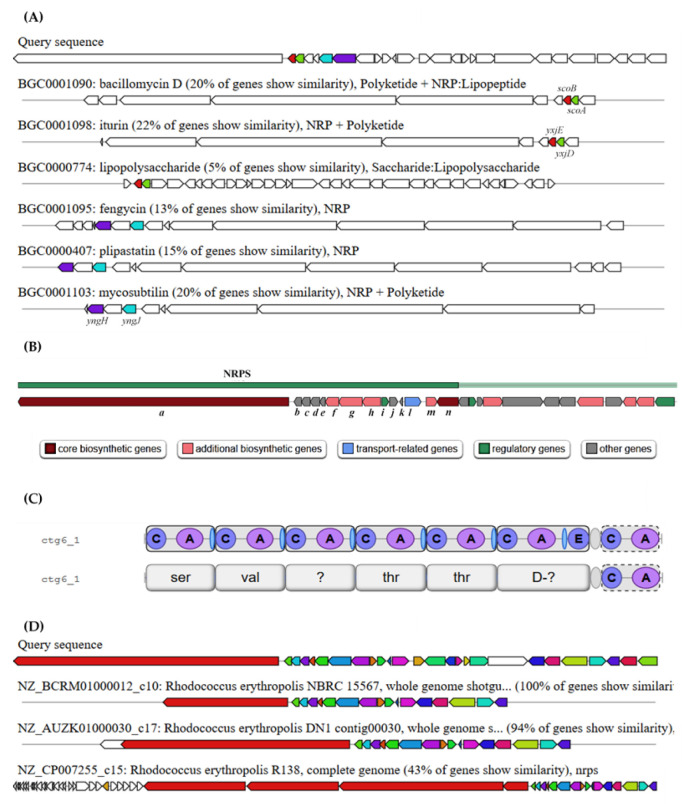
The gene clusters predicted by antibiotics and secondary metabolite analysis shell (anti-SMASH) for the *Rhodococcus* sp. ADL36 draft genome. (**A**) Homologous known gene clusters; (**B**) schematic representation of the assembly line for the lipopeptide-encoding NRPS-6; (**C**) anti-SMASH prediction of principal locus in NRPS-6 (with and without module domain); (**D**) homologous gene clusters.

One NRPS cluster (NRPS-6) was revealed to have 22, 20 and 20% similarity to iturin, bacillomycin D and mycosubitilin, respectively, which are all members of the iturinic lipopeptides (Figure 2A). A classic iturin family is a cyclic lipoheptapeptides connected via a *β*-amino acid residue. The members of that family commonly exhibit strong antibiotic and moderate surfactant activities [57]. Though the similarity of the predicted NRPS was skewed towards the iturinic lipopeptide, the resemblances were only through the subsidiary biosynthetic genes of the lipopeptide—bacillomycin D (*scoA*, *scoB*); iturin (*yxjD*, *yxjE*); and mycosubtilin (*yngH*, *yngJ*). Besides, no open reading frames (ORFs) of bacillomycin D (*bam*/*bmy*), mycosubtilin (*myc*) or iturin (*itu*) were found in the cluster. However, it has to be kept in mind that the aforementioned features of the *C*-starter domain as the key character for the synthesis of lipopeptides still holds true. The special starter domain positioned at the *N*-terminal of the first module implies that the first substrate is initially *N*-acylated with a *β*-hydroxy fatty acid [57,58]. The hydrophobic enhancement of the hydrophilic NRP products by *N*-acylation can significantly contribute to the variation of peptide sequences [59]. The distinctive *N*-acylated *C*-starter domain is a key structural element in well-known lipopeptide surfactants such as lichenysin [60], fengycin [61], surfactin and arthrofactin [58].

Though in silico prediction of the lipopeptide NRPS might encode for either antibiotic or biosurfactant, the stark homogeneity in the assembly lines of the NRPS-6 region as lipopeptide biosurfactants was more relevant and significant (Figure 2B). This is due to the succession of encrypted acyl/acetyl-CoA homologous gene participating for the generation of fatty acid precursor, typically observed in the lipopeptide biosurfactant’s NRP assembly lines [56,57,58]. From the figure, ORF *a* denotes the main module which consists of the essential catalytic domain. The gene homologs for the fatty acid synthesis can be observed from ORFs *h* to *b*. The predicted *h* and *g* encode for the acetyl-CoA carboxylase *β*- (*accD*) and *α*-subunit (*accA*, *yngH* analog), respectively. These genes carboxylate acetyl-CoA to a malonyl-CoA building block, a significant substrate for initiating the de novo biosynthesis of fatty acids [62]. ORF *f* denotes for the enzyme acyl-CoA dehydrogenase (ACAD) which catalyses the *β*-oxidation of branched-chain and unsaturated fatty acids [63]. The dehydrogenase complex may also take part in the conversion of the CoA precursors to the respective fatty acid chains [64]. ORFs *e* and *d* code for the MaoC family dehydratase and CoA ester lyase, respectively. The MaoC-like dehydratase domain is analogous to the fatty acid synthase *β*-subunit [65]. Finally, the ORFs *c* and *b* are predicted to be the putative homologs for the CoA transferase subunit A (*scoA*/*yxjD*) and B (*scoB*/*yxjE*). Although the subunits have been predicted to be involved in certain processes such as oxidative stress response [66], their role in the production of lipopeptide is more likely due to their significant homology towards the CoA transferases modules in the assembly lines of iturin [67] and bacillomycin [68]. The biosynthetic ORFs *n* and *m* are believed to encode for acyl-CoA synthetase and glycosyltransferase family 2 protein, respectively. Nevertheless, the non-sequential enzymatic order for fatty acid metabolism causes the assembly lines of the NRP to contradict with the customary order observed in most lipopeptide biosurfactant biosynthetic pathways. This unusual architecture of modules have the potential for the establishment of a new fatty acid synthase complex owed to the presence of the above-mentioned genetic ORFs.

The main module (a) of NRPS-6 consisted of six complete modules (a module encodes an amino acid) and an incomplete module with the predicted structure of FA-β-OH-L-Ser-L-Val-L-X-L-Thr-L-Thr-D-X-_(?)_X (Figure 2C). Consecutive analysis via antiSMASH-integrated NRPSpredictor2 [69] displayed a much-improved prediction of the module with a structural estimation of FA-β-OH-L-Ser-L-Val-L-[Val/Leu]-L-Thr-L-Thr-D-Leu-_(?)_Ser. The D-amino acid at the sixth module was deduced because of the presence of an epimerization (E) domain which stereochemically converts a former L-isomer. From the predicted region of ORF, two notable features can be observed in the last module. Firstly, the module is shown to be incomplete as it does not contain a thiolation (T/PCP) domain, one of the three catalytic domains in the NRPS linear assembly. With the absence of the PCP, the predicted residue that would be loaded is not regarded as the candidate for the cluster’s polymer prediction and the module ought to be considered as a partial module. Secondly, the lack of TE domain in the last module is confounding. Widely known as the termination domain, it catalyzes the cyclisation and release of a complete peptide residue. The domain is generally located at the end of an NRPS assembly line and is comprised of conserved catalytic substrates required for product release [57]. The absence of the TE domain has been reported formerly in the fungal NRPS assembly line where variants of terminal C domains act as the termination domain [70,71]. However, the lack of a TE domain in the last module in this study cannot be hypothesized as a novel biosynthetic pathway for a lipopeptide biosynthesis. This assertion is based on the prediction of BGCs through a draft genome which highly relies on the segments of contigs. A draft genome covers contig fragments which are interspersed with gaps that contain unknown sequences. These gaps signify the loss of genomic data which frequently corresponds with critical genomic loci. It may also deter the prediction of a complete NRPS (or lipopeptide synthetases) as unresolved genes and their incomprehensible regulations are limiting the true analysis.

From Figure 2D, the only complete genome that shows homologous gene clusters and layouts to ADL36 is *R. erythropolis* R138. The genome consists of four major modules, rich in serine residues and containing a canonical TE domain at the last module. While the function of the homologous NRPS is yet to be studied, the presence of ORFs for lipase and esterase (which generally correlate to the biosurfactant production) in R138 might indicate the synthesis of this substrate.

Although *Rhodococcus* spp. are well-known biosurfactant producers in temperate environments [31,32], the ability to synthesise surface-active agents is recognised as a general feature among cold-adapted microorganisms from this phylotype [4,5]. Perfumo et al. [9] suggested that the ecological role of biosurfactants in Antarctica is to facilitate the indigenous microbial communities in metabolising plant-derived substances which are naturally enriched in organic hydrocarbon and aromatic compounds. It also facilitates the production of extracellular enzymes and promotes the deterioration of hydrocarbon pollutants. They also suggested that biosurfactants could play a crucial ecological role in the turnover of carbon and nutrients in extremely cold soils. This role is likely owed to their synergistic effect with the extracellular enzymes to enhance the solubility and bioavailability of the hydrophobic residues.

The best-described biosurfactants amongst the *Rhodococcus* genus are the glucose-based glycolipids, called trehalolipid [13,14]. This is related to the high mycolic acid (mycolate) content in the Rhodococci cell wall where most elucidated trehalose-comprising glycolipids carry a mycolic fatty acid ester as their hydrophobic moiety [13]. However, the specificity of a synthesised biosurfactant can still depend on the utilisation of the substrate and the nature of the biosurfactant itself. Rhodofactin synthesised by the oil-degrading *Rhodococcus* sp. TW53 was the first account of a lipopeptide-producing *Rhodococcus* [15], although several lipopeptides have been reported to be synthesised from the class of Actinobacteria [13]. Up until now, only two surface-active lipopeptides synthesised by the genus *Rhodococcus* have been reported (Table 3). According to Peng et al. [15], the absence of glycolipids in the sample was not related to the extraction method, but rather to the lack of corresponding synthetic pathways in the bacterium itself. A genome-based study by Ceniceros et al. [53] on the metabolic capacities of the genus *Rhodococcus* revealed a great number of putative BGCs in their genome which includes a considerable number of BGCs that putatively encode the lipopeptide synthesis. This genomic exploration of the Rhodococci genome provides the base for further research focusing on the assessment of biosurfactant production, particularly the less-studied Rhodococci lipopeptides.

To date, the number of known cold-adapted and biosurfactant-producing *Rhodococcus* strains is still very low (Table 4) and their respective biosurfactant is not well-studied in terms of characterisation, structural elucidation and high-scale production. Kennicutt et al. [73] believed that this under-explored chemical and microbial biodiversity of the polar zones may reflect the logistical challenges and limited studies rather than the real situation.

While the discovery of the lipopeptide-producing *Rhodococcus* from the Antarctic can unlock new possibilities and prospects in various biotechnological applications, the dual reputation of ADL36 as a hydrocarbon-degrading and biosurfactant-producing bacterium should be emphasised within the context of Antarctic soil remediation. To begin with, the remediation of hydrocarbon-polluted Antarctic soil requires the involvement of the native Antarctic populations as the Antarctic Treaty outlaws the introduction of non-indigenous microorganisms [1]. Owing to the extreme Antarctic conditions and augmented by the resistant characteristics of hydrocarbon substrates, the pollution in both soil and groundwater systems may pose a long-term risk to these pristine ecosystems. The utilisation of biosurfactants in Antarctic soil remediation has advantages over the commonly-used chemical surfactants due to their low environmental effects and solubilisation capability towards hydrocarbon pollutants. The combination of in situ production of the biosurfactants via biostimulation and bioaugmentation would also contribute to a faster clean up rate. Biosurfactant-boosted soil remediation studies such as the smaller scale preliminary simulated experiment with controlled environmental conditions (that met the Antarctic cold settings), to larger scale pilot studies should be continuously researched and applied deliberately. This broadens the paucity of options for in situ remediation efforts in the Antarctic which have to follow the strict requirements of the Annex III of the Protocol on Environmental Protection to the Antarctic Treaty (Madrid Protocol) [74].

## 3. Materials and Methods

### 3.1. Chemical and Reagents

Diesel fuel (PETRONAS Genrail5, SAE 40) used throughout the experiment was purchased from the local gas station (PETRONAS, Malaysia). Diesel was selected as the model pollutant because it is considered to be a generic environmental noxious waste. Used motor oil (Liqui Moly, Motorbike 10 W-40 Street, Ulm, Germany) was obtained from the local automobile repair shop near the gas station. The dark-coloured used motor oil was used along with diesel in the emulsification capacity test while only used motor oil was used in oil displacement tests to compare and attain a more distinct observation. All the other reagents, chemicals and biochemicals used in this report were of analytical grade.

### 3.2. Microorganism

The hydrocarbon-oxidising microbial strain used in this study was isolated from the Antarctic fellfield soils collected in coastal environments of southern Victoria Land [77]. Prior to isolation processes, the soils were enriched with diesel fuel as the sole carbon and energy source for the selection of an appropriate bacterial strain. The strain was identified according to several methods described in Bergey’s Manual of Determinative Bacteriology and via 16S rDNA sequence analysis. The isolate was designated as *Rhodococcus* sp. ADL36 and assigned under the accession number of KX812777 [10]. The isolate was stored at −80 °C in Bushnell Haas (BH) medium supplemented with 80% (*v*/*v*) glycerol solution.

### 3.3. Media and Growth Conditions

Two different enrichment culture media were used throughout the experiment: nutrient broth and BH medium. Nutrient broth acts as the medium for cell suspension preparations. BH medium was used to examine microbial hydrocarbon utilisation and for biosurfactant production. The BH medium was composed of (per litre of solution) 0.2 g of MgSO_4_, 0.02 g CaCl_2_, 1.0 g K_2_HPO_4_, 1.0 g KH_2_PO_4_, 1.0 g NH_4_NO_3_ and 0.05 g FeCl_3_. The pH of the media was adjusted to 7.0 ± 0.2 at 25 °C prior to sterilisation at 121 °C for 15 min. The isolate was initially grown in 100 mL of nutrient broth for 48 h prior to cell cultivation. Culture was grown in nutrient broth (20 °C, 150 rpm), centrifuged at 10,000 rpm for 20 min, washed with 1X phosphate-buffered saline (PBS: 137 mM NaCl, 2.7 mM KCl in 10 mM phosphate buffer, pH 7.4) solution and the cell density was finally adjusted to OD_600_ nm = 1. For the evaluation of the Antarctic isolate to produce biosurfactant, aliquots (1 mL) of bacterial suspension was used to inoculate 100 mL of BH media supplemented with 1.0% (*v*/*v*) diesel as a sole carbon source. All trials were performed in a 250 mL conical flask shaken at 150 rpm for 3 days. The initial value for pH and temperature during the fermentation was put at pH 7.0 and 20 °C, respectively. All assays were performed in triplicates.

### 3.4. Screening of Bacteria for Biosurfactant Production

The production of biosurfactants by isolate was examined using several qualitative methods such as BATH assay, drop collapse test, oil displacement activity, microplate assay and emulsification capacity test. The cultures were initially centrifuged (10,000 rpm, 20 min at 4 °C), and only the cell-free supernatants were used with the exclusion of BATH assay.

#### 3.4.1. Hydrophobicity Assay

The relative hydrophobicity of strain ADL36 cells was measured by BATH assay according to Rosenberg et al. [23] with slight modification. The cells were harvested via centrifugation (10,000 rpm, 20 min at 4 °C) and washed twice with 50 mM K_2_HPO_4_ (pH 6.5) buffer. They were then resuspended using the same buffer to OD_600_ nm = 0.5. 3 mL of cell suspensions which were added to tested hydrocarbons (0.5 mL) and vortexed for 3 min. The mixture was then allowed to separate from the hydrocarbon and aqueous phase for 45 min. The aqueous phase was removed and measured at 600 nm [23,24]. The reduction in the absorbance of the aqueous phase was taken as a measure of the cell-surface hydrophobicity (*H%*), which was calculated according to Equation (1):

*H%* = [(*A*_0_ − *A*)/*A*_0_] × 100 (1)

where *A*_0_ and *A* were absorbance values before and after the addition of tested hydrocarbons, respectively.

#### 3.4.2. Drop Collapse Test

A drop collapse test was carried out according to both Bodour and Miller [26] and Youssef et al. [27] procedure with slight modification. A sterile glass slide surface was coated with a thin layer of diesel oil. The coated glass surface was equilibrated for 24 h to ensure a uniform oil coating. 10 µL of cell-free supernatant was pipetted onto the surface of a coated glass slide and left for an hour. The experiment was conducted in triplicates. For qualitative observation, a flat shape drop indicated the presence of biosurfactant while a rounded drop signified the absence of biosurfactant.

#### 3.4.3. Oil Displacement Test

For oil displacement activity, 20 mL of distilled water was poured into a clean petri dish (85 mm diameter). 300 µL of oil sample was subsequently added to the surface of the water until an even and thin layer of oil was established. Then, an equal volume of cell-free supernatant was dropped onto the oil surface. The formation of a clear zone as the indication for the presence of a biosurfactant was then observed qualitatively. Distilled water was employed as the negative control while both SDS and Triton X-100 were used as positive control.

#### 3.4.4. Qualitative Microplate Analysis

The surfactant activity of the Antarctic strain was determined qualitatively using a 96-microwell plate assay. 100 µL supernatant was added to the microwell of a 96-microwell plate. The plate was then viewed using a backing sheet of a paper with a black and white grid. The optical distortion of the grid provided a qualitative assay for the presence of a surfactant [29]. Distilled water was used as the control.

#### 3.4.5. Emulsification Capacity Assay

The emulsification capacity was determined by the addition of an equal volume of cell-free supernatant to 2 mL of diesel oil and used motor oil. The mixture was then vortexed at high speed for 2 min and left to stand for 24 h [78]. The E_24_ was calculated as the ratio of the height of the emulsion layer to the total height of the liquid presented using Equation (2):

E_24_ (%) = (*h* emulsion/*h* total) × 100 (2)

where E_24_ is the emulsion index after 24 h, *h* emulsion is the height of the emulsion layer, and *h* total is the height of liquid.

### 3.5. Determination of Surfactant Stability

#### 3.5.1. Biosurfactant Stability Test

The biosurfactant stability was investigated using the E_24_ and ST test. Both were conducted with respect to the effect of temperature, pH and salinity on the surface tension. Furthermore, the correlation between the ST measurements and the emulsification capacity of the biosurfactant was assessed on the thermal, pH and salinity stability. The cell-free broth was obtained from a 24 h culture via centrifugation (10,000 rpm, 20 min at 4 °C). The stability against pH was assessed by modifying the pH of 20 mL supernatant from 5.0 to 9.0 using 1 M HCl or 1 M NaOH solution. To test the effect of temperature, the supernatant was subjected to different temperatures (15, 20, 25, 30, 35 °C) in an incubator for 15 min. The effect of NaCl concentration was studied by the addition of the increasing amount of NaCl (*w*/*v*) (3%, 6%, 9%, 12%) into the supernatant. For 0% of NaCl stability assay, no NaCl was added into the cell-free broth. All samples were allowed to stand at room temperature for 6 h after respective treatments prior to the evaluation of ST and E_24_.

#### 3.5.2. Surface Tension Measurements

The ST values were verified with Sigma 700 digital tensiometer (Attension, Espoo, Finland). The tensiometer was calibrated using the ST of Milli-Q water (72 mN/m). The measurements of the ST were executed using the du Noüy ring method [30]. Each evaluation of STs was performed in triplicates at ambient temperature. A sample of culturing medium without any bacterial inoculation was applied as the control. The results were reported as mean and standard deviation (SD) values of repeated measurements. Cell-free supernatant solution (20 mL) was poured into a clean 50 mL glass beaker and placed onto the tensiometer platform. The ring was then submerged into the solution and slowly pulled through the liquid–air interface. The platinum ring was rinsed thrice with water and acetone, flamed with a blowtorch and allowed to cool to remove any surfactant residues. ST lower than 40 mN/m can be considered as an expression of biosurfactant production [26,79].

### 3.6. Extraction of Biosurfactant

The extraction process was performed to obtain the crude biosurfactant for more distinctive analysis through acid precipitation. Bacterial cells were removed via centrifugation at 10,000 rpm (30 min at 4 °C). The culture supernatant was acidified with 6 N HCl to bring the final pH to 2.0 for overnight incubation. The extraction was initiated by the addition of an equal volume of two different solvent systems. The solvent systems used were MTBE [80] and chloroform:methanol (2:1). The mixture was then shaken on a rotary shaker (200 rpm) for 24 h and left to stand until phase separation. The solvent layer was separated from the aqueous phase, and the product was concentrated by a rotary vacuum evaporator at 50 °C under reduced pressure [80]. The crude (partially purified) extract was lyophilised after solvent evaporation for further characterisation.

### 3.7. Carbohydrate, Protein and Lipid Estimation

The sugar content in the extracted biosurfactant molecules was assessed using phenol-sulphuric assay according to the method proposed by DuBois et al. [81] with glucose (0.1 mg/mL) as the standard sugar. The presence of peptide groups in the biosurfactant was determined by the Lowry test [82]. Bovine serum albumin (BSA) (1000 µg/mL) was used to construct the calibration curve, and the absorbance of the sample was measured at 660 nm. The lipid content was extracted from the chloroform layer by means of liquid–liquid extraction following Folch et al. [83].

### 3.8. Thin-Layer Chromatography Analysis

Partially purified extract (100 mg) was dissolved in an appropriate solvent and subjected to TLC silica gel plates (60 *F*_254_ Merck). The TLC fractionation was conducted for the separation of biomolecules with the following solvent system: chloroform:methanol:water (65:35:4) for lipids, chloroform:acetic acid:water (50:30:20) for carbohydrates and *n*-butanol:acetic acid:water (4:1:1) for proteins/amino acids. The detection of lipid fractions was done through TLC plate exposure to iodine vapour while the visualisation of carbohydrates and amino acids spots were acquired by the spraying of Molisch reagent and ninhydrin solution (1%), respectively. After exposure, sprayed TLC plates were heated at 110 °C for 30 min until the appearance of respective colour.

### 3.9. Infrared Analysis

The FTIR spectroscopic analysis was performed on a Spectrum^TM^ 100 1FTIR spectrometer (Perkin Elmer, Waltham, MA, USA) with the 5 mg lyophilised sample dispersed in pellets of potassium bromide (KBr) to elucidate the types of chemical bonds (functional groups). The spectral measurements were carried out in the range 400–4000 cm^−1^ wave number. All data were corrected for the background spectrum using KBr pellets. The functional groups of biosurfactants were detected therefore revealing the chemical nature.

### 3.10. DNA Extraction, Genome Sequencing and Annotation

The *Rhodococcus* sp. ADL36 genomic DNA was isolated using DNeasy^®^ Blood and Tissue Kit (Qiagen, Heilden, Germany) in accordance with the manufacturer’s procedure and protocol. Nextera XT DNA libraries were prepared following the manufacturer’s protocol (Illumina, San Diego, CA, USA). Genome sequencing was performed on the Illumina MiSeq platform (Illumina, San Diego, CA, USA) using a 500 cycle MiSeq Reagent kit v2 (MS-102-2003, Illumina, San Diego, CA, USA) by means of both sequencing by synthesis (SBS) and paired-end 2 × 251 sequencing. Paired-end reads were subjected to quality filtering and de novo genome assembly using both St. Petersburg genome assembler (SPAdes) [84] and Velvet assembler [85]. The genome assembly was annotated using the Rapid Annotations using Subsystems Technology (RAST) server [86]. The rRNA genes were predicted using RNAmmer [87] while the tRNA genes were predicted using ARAGORN [88]. The protein coding genes were first predicted using prokaryotic dynamic programming gene-finding algorithm (Prodigal) [89], and the predicted sequences were used to predict their function by using Basic Local Alignment Search Tool (BLAST) [90] and HMMER [91] to search against various sequence or domain databases.

### 3.11. Secondary Metabolites Analysis

The draft genome sequence for *Rhodococcus* sp. ADL36 was analyzed by the anti-SMASH version 5.0 (bacterial version) (https://antismash.secondarymetabolites.org) with the selection of all extra annotation features and strict detection [92].

## 4. Conclusions

The current work characterizes the *Rhodococcus* sp. ADL36, an Antarctic hydrocarbon degrader, to produce biosurfactants. Positive screening of biosurfactant production was observed for both surface/interfacial activity and cell-surface hydrophobicity. Excellent stability for the supernatant was displayed within the whole range of all the examined variables. An incomplete correlation was observed between E_24_ and surface activity of the cell-free supernatant. TLC analysis and FTIR spectroscopy implied that the biosurfactant is a lipopeptide, although further purification and structural characterization should be conducted to reveal the precise composition of the Antarctic lipopeptide. The genome excavation on ADL36, which discloses a significant amount of putative BGCs that includes the synthesis of surface-active lipopeptides, strengthens the prospect of novel natural products. However, improved sequencing (genome completeness and higher quality) may increase the understanding of the biosurfactant production. Application-wise, the strain ADL36 and its lipopeptide have major potential for hydrocarbon removal in Antarctic soil. This new and rare occurrence of lipopeptide biosurfactant in Rhodococci may also entice interest from the community regarding natural products research in exploring the often overlooked actinomycetes.

## Figures and Tables

**Table 1:** General features of the genomes of *Rhodococcus* species that are closely annotated to *Rhodococcus* sp. ADL36.

Strain	*Rhodococcus* sp. ADL36	*R. erythropolis* PR4 [49]	*R. erythropolis* CCM2925 [51]
Genomic organization			
Genomic size (Mb)	8.44	6.89	6.37
G + C (%) ^1^	63.28	62.29	62.50
Number of CDS ^2^	8462	6293	5743
tRNAs	82	54	53
rRNA gene clusters	6	15	12
Assembly and annotation level	Contigs (draft)	Complete (whole)	Complete (whole)
Isolate origin	Antarctic soil	Pacific Ocean	Unspecified soil

^1^ Guanine-cytosine content; ^2^ Number of coding sequences (protein-coding genes).

**Table 2:** The distribution of biosynthetic gene clusters in the *Rhodococcus* sp. ADL36.

Region	Cluster Type	Gene Cluster(s)	Description	Similarity (%)
1.1	NRPS	Heterobactin A/S2	NRP ^1^	100
2.1	Bacteriocin	Branched-chain fatty acids	Other	75
2.2	Linear azol(in)e-containing peptides	Diisonitrile antibiotic SF2768	NRP	11
2.3	T1PKS ^2^	—	—	—
3.1	NRPS	Erythrochelin	NRP	57
4.1	NRPS	—	—	—
4.2	NRPS	Monensin	Polyketide	5
4.3	Terpene	Isorenieratene	Terpene	42
4.4	Ectoine	Ectoine	Other	75
6.1	NRPS	Bacillomycin D, iturin, mycosubtilin	Polyketide + NRP: lipopeptide	20, 22, 20
8.1	NRPS	Coelichelin	NRP	27
8.2	NRPS, terpene	SF2575	Polyketide: type II + saccharide: hybrid/tailoring	6
9.1	Lanthipeptide	—	—	—
10.1	Butyrolactone	—	—	—
10.2	Aminoglycoside/aminocyclitol cluster	Acarbose	Saccharide	7
13.1	NRPS	Rifamorpholine A–E, rifamycin	Polyketide	4, 4
67.1	NRPS	—	—	—
181.1	Lasso peptide	—	—	—

^1^ Non-ribosomal peptide; ^2^ Type 1 polyketide synthase.

**Table 3:** List of surface-active lipopeptide-producing bacteria from the genus *Rhodococcus*.

Strain(s)	Origin	Enrichment Substrate	ST (mN/m) ^1^	E_24_ (%) ^2^	Surfactant Content (%)	Reference(s)
*Rhodococcus* sp. TW53	Deep-sea sediment, west Pacific Ocean	*n*-hexadecane	34.4 (S) ^3^, 30.7 (P) ^4^	*n*/a ^5^	*n*.d ^6^	[15]
*R. ruber* MP4	Soummam sediments, Bridge Skala of Bejaia, Algeria	Petroleum + glucose	*n*.d	78.50 (S), 87.77 (P)	Protein: 10.5; lipid: 64.2	[16,72]
*Rhodococcus* sp. ADL36	Fellfield soils, coastal ice-free environments, Southern Victoria Island, Antarctica	Diesel	29.7	45.33	Protein: 25.0; lipid: 64.0; sugar: < 3.0	This study

^1^ surface tension measurement; ^2^ emulsification index; ^3^ supernatant; ^4^ purified biosurfactant; ^5^ not available; ^6^ not determined.

**Table 4:** List of polar hydrocarbon-degrading *Rhodococcus* strains that synthesize surface-active agents.

Strain(s)	Origin	Substrate	Biosurfactant Produced	ST (mN/m) ^1^	E_24_ (%) ^2^	T (°C) ^3^	Reference(s)
*Rhodococcus* spp.							[5]
176; 179; 181; 187	South Shetlands Islands (Antarctica) and Svalbard Islands (Norwegian Arctic)	Tetradecane	Glycolipid	28.3; 27.3; 28.6; 28.2	58; 37; 33; 57	4; 15	
*R. fascians* A-3	Casey Station, Wilkes Island, Antarctica	Kerosene	Rhamnose-containing lipid	27	*n*.d ^4^	28	[4]
*R. fascians* BD8	Arctic Archipelago of Svalbard	*n*-hexadecane	Trehalose lipid	34–36	*n*.d	28	[75]
*Rhodococcus* sp.	Terra Nova Bay (Ross Sea, Antarctica)	Aliphatic/aromatic hydrocarbons	Trehalose lipid	32	*n*.d	*n*/a ^5^	[76]
*Rhodococcus* sp. ADL36	Fellfield soils, coastal ice-free environments, Southern Victoria Island, Antarctica	Diesel	Lipopeptide	29.7	45.3	20	This study

^1^ surface tension measurement; ^2^ emulsification index; ^3^ temperature; ^4^ not determined; ^5^ not available.

## Supplementary Materials

The following are available online at https://www.mdpi.com/1422-0067/21/17/6138/s1.

## Abbreviations

BATH	Bacterial adhesion to hydrocarbon
BGC	Biosynthetic gene cluster
BH	Bushnell Haas
BLAST	Basic Local Alignment Search Tool
BSA	Bovine serum albumin
FTIR	Fourier-transform infrared
MTBE	Methyl-tert-butyl ether
NRPS	Non-ribosomal peptide synthetase
ORF	Open reading frame
PBS	Phosphate-buffered saline
PCP	Peptidyl carrier protein
PKS	Polyketide synthase
RAST	Rapid Annotations using Subsystems Technology
ST	Surface tension
TE	Thioesterase
TLC	Thin-layer chromatography
