# Supplementary material for: Production of Lipopeptide Biosurfactant by a Hydrocarbon-Degrading Antarctic Rhodococcus
Source: Int J Mol Sci. 2020 Aug 26;21(17):6138. doi: 10.3390/ijms21176138 (PMC7504157; doi:10.3390/ijms21176138)
Supplement: Supplementary file 1 [file ijms-21-06138-s001.pdf]

International Journal of *Molecular Sciences*

MDPI

Article

Production of Lipopeptide Biosurfactant by a Hydrocarbon-Degrading Antarctic *Rhodococcus*

Syahir Habib [1], Siti Aqlima Ahmad [1], Wan Lutfi Wan Johari [2], Mohd Yunus Abd Shukor [1], Siti Aisyah Alias [3], Jerzy Smykla [4], Nurul Hani Saruni [1], Nur Syafiqah Abdul Razak [1] and Nur Adeela Yasid [1,*]

[1] Department of Biochemistry, Faculty of Biotechnology and Biomolecular Sciences, Universiti Putra Malaysia, Serdang, Selangor 43400, Malaysia; syahirhabib@gmail.com (S.H.); aqlima@upm.edu.my (S.A.A.); mohdyunus@upm.edu.my (M.Y.A.S.); hanisaa17@gmail.com (N.H.S.); syaffy27@gmail.com (N.S.A.R.)

[2] Department of Environment, Faculty of Forestry and Environment, Universiti Putra Malaysia, Serdang, Selangor 43400, Malaysia; wanlutfi@upm.edu.my

[3] Institute of Ocean and Earth Sciences, C308 Institute of Postgraduate Studies, University of Malaya, Kuala Lumpur 50603, Malaysia; saa@um.edu.my

[4] Institute of Nature Conservation, Polish Academy of Sciences, Mickiewicza 33, 31-120 Kraków, Poland; jerzysmykla@yahoo.com

* Correspondence: adeela@upm.edu.my; Tel.: +603-9769-8297

Received: 21 July 2020; Accepted: 8 August 2020; Published: 26 August 2020

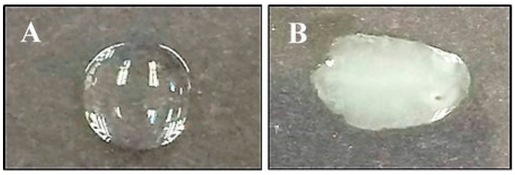

Figure S1. The drop-collapse test of bacterial supernatant of ADL36. (**A**) The droplet shape of distilled water (control) remains stable while a near-complete collapse was observed for the supernatant (**B**).

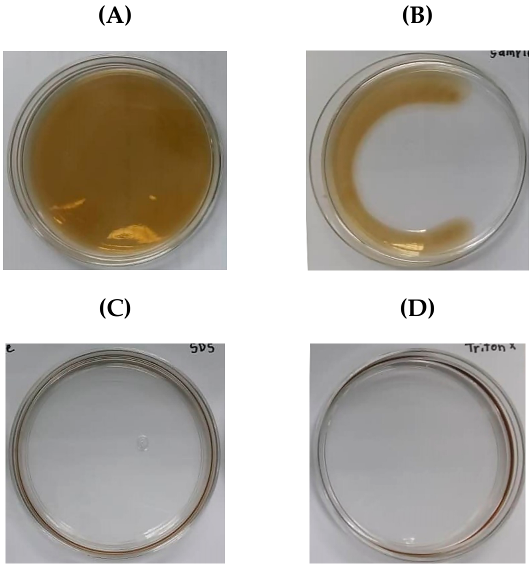

Figure S2. The illustration of oil displacement activity for (**A**) distilled water; (**B**) bacterial supernatant of ADL36; (**C**) 1.0% sodium-dodecyl-sulphate (SDS); and (**D**) 1.0% Triton X-100.

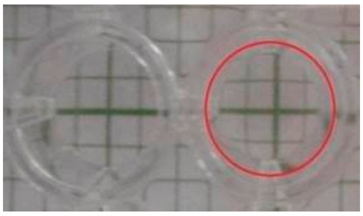

Figure S3. The qualitative assessment of biosurfactant production by 96-well microplate assay for *Rhodococcus* sp. ADL36. The right well (**red circle**) filled with bacterial supernatant showed distortion and appeared smaller than the right well (distilled water) indicating the presence of surface-active agents. The control (distilled water) showed a flat image.

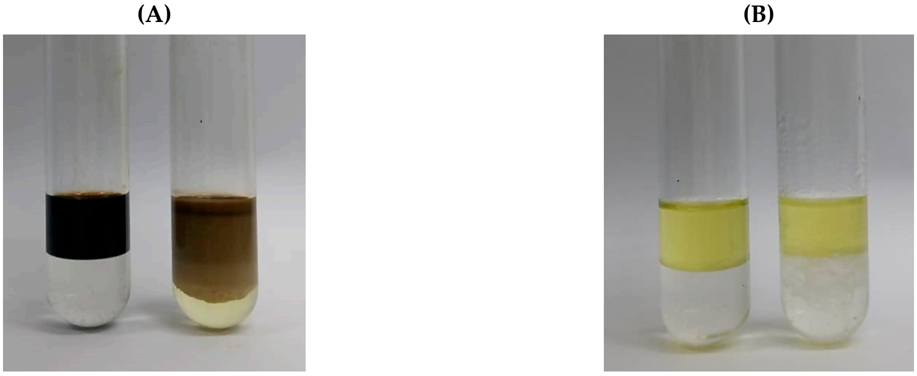

Figure S4. The emulsification of oil with cell-free supernatant using (**A**) used motor oil and (**B**) diesel oil. Distilled water (left) was used as a control in both experiments showed a distinct separation of two layers. Formation of emulsion layer (in both test tubes on the right) can be observed with the addition of supernatant from ADL36.

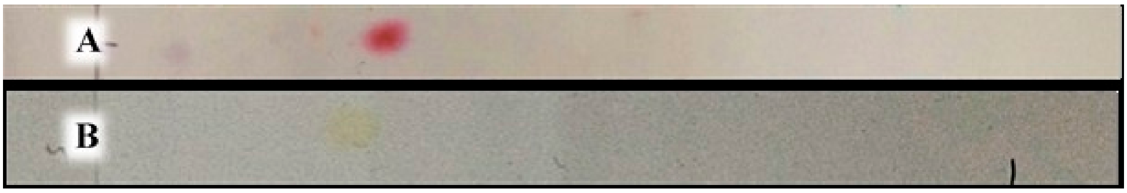

Figure S5. The formation of coloured spots on the thin-layer chromatography (TLC) plate through the exposure to ninhydrin (**A**) and iodine vapour (**B**).

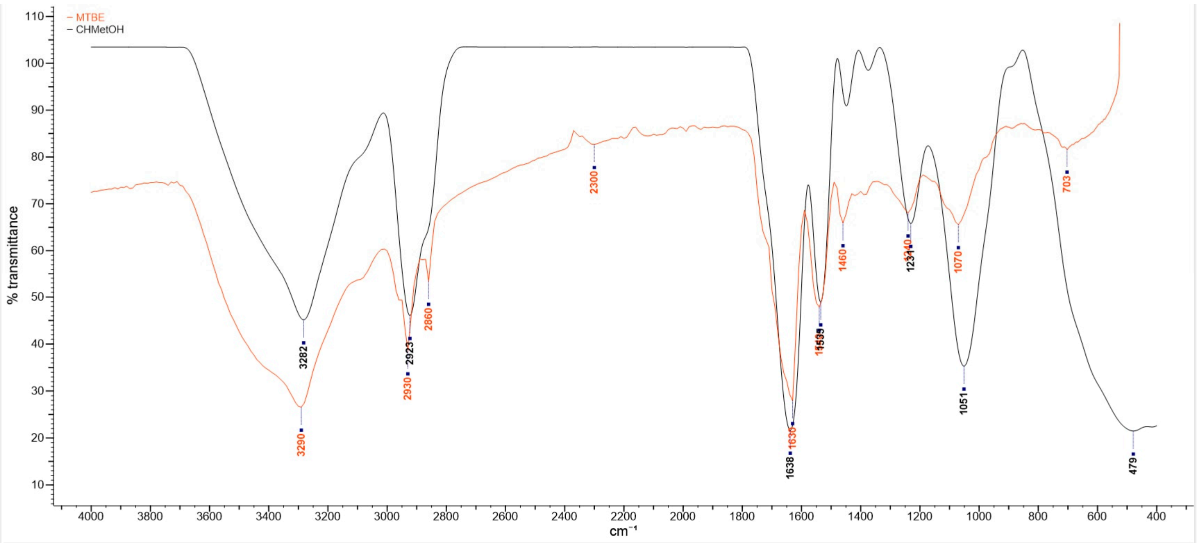

Figure S6. The FTIR spectra for the biosurfactant produced by *Rhodococcus* sp. ADL36. The red line indicates the biosurfactant extracted using methyl-tert-butyl ether (MTBE) while the black line indicates the biosurfactant extracted by chloroform-methanol solvent system.

© 2020 by the authors. Licensee MDPI, Basel, Switzerland. This article is an open access article distributed under the terms and conditions of the Creative Commons Attribution (CC BY) license (http://creativecommons.org/licenses/by/4.0/).
